# The dysfunctional immune response in renal cell carcinoma correlates with changes in the metabolic landscape of ccRCC during disease progression

**DOI:** 10.1007/s00262-023-03558-5

**Published:** 2023-11-08

**Authors:** Nicola E. Annels, M. Denyer, D. Nicol, S. Hazell, A. Silvanto, M. Crockett, M. Hussain, Carla Moller-Levet, Hardev Pandha

**Affiliations:** 1 Oncology, Department of Clinical and Experimental Medicine, University of Surrey, Guildford, UK; 2 Royal Marsden Hospital, Fulham Road, London, UK; 3 Frimley Park Hospital, Frimley, Camberley, UK; 4 Bioinformatics Core Facility, University of Surrey, Guildford, UK

**Keywords:** Renal Cell carcinoma, T-cell exhaustion metabolic

## Abstract

Renal cell carcinoma is an immunogenic tumour with a prominent dysfunctional immune cell infiltrate, unable to control tumour growth. Although tyrosine kinase inhibitors and immunotherapy have improved the outlook for some patients, many individuals are non-responders or relapse despite treatment. The hostile metabolic environment in RCC affects the ability of T-cells to maintain their own metabolic programme constraining T-cell immunity in RCC. We investigated the phenotype, function and metabolic capability of RCC TILs correlating this with clinicopathological features of the tumour and metabolic environment at the different disease stages. Flow cytometric analysis of freshly isolated TILs showed the emergence of exhausted T-cells in advanced disease based on their PD-1^high^ and CD39 expression and reduced production of inflammatory cytokines upon *in vitro* stimulation. Exhausted T-cells from advanced stage disease also displayed an overall phenotype of metabolic insufficiency, characterized by mitochondrial alterations and defects in glucose uptake. Nanostring nCounter cancer metabolism assay on RNA obtained from 30 ccRCC cases revealed significant over-expression of metabolic genes even at early stage disease (pT1-2), while at pT3-4 and the locally advanced thrombi stages, there was an overall decrease in differentially expressed metabolic genes. Notably, the gene *PPARGC1A* was the most significantly down-regulated gene from pT1-2 to pT3-4 RCC which correlated with loss of mitochondrial function in tumour-infiltrating T-cells evident at this tumour stage. Down-regulation of PPARGC1A into stage pT3-4 may be the ‘tipping-point’ in RCC disease progression, modulating immune activity in ccRCC and potentially reducing the efficacy of immunotherapies in RCC and poorer patient outcomes.

**Supplementary Information:**

The online version contains supplementary material available at 10.1007/s00262-023-03558-5.

Background

In the UK, renal cell carcinoma (RCC) is the fifth most common malignancy in men and the tenth in women [1]. Risk factors include hypertension, smoking, obesity and end-stage renal disease. A third of cases present with metastatic disease at diagnosis and a third of those undergoing surgery for early stage disease will relapse, after a median 1.9 years [2]. RCC has joined the growing list of cancers where the PD-1 immune checkpoint inhibitor nivolumab alone or in combination with ipilimumab [3] has improved overall survival, resulting in a reappraisal of immunotherapy for RCC treatment [4]. Recent studies have shown checkpoint inhibitor combination with tyrosine kinase inhibitors (TKIs) leads to higher response rates still, which are durable and have improved patient survival [5]. Although a number of IO/TKI combinations are currently being utilized in the clinic with impressive response rates, the mechanisms underlying the synergistic effects are unknown, particularly that alternative TKIs have differing immunoregulatory and metabolic effects. There is a paucity of studies addressing the T-cell or tumour metabolic consequences of TKI exposure in combination with immunotherapy. Among the most compelling data highlighting the immunoregulatory potential of TKIs is that associated with Lenvatinib and enhanced T-cell infiltration and proliferation as well as a reduction in myeloid derived suppressor cells in the TME [6].

RCC is considered an immunogenic tumour with a prominent dysfunctional immune cell infiltrate, unable to control tumour growth [7, 8]. Although effector T-cells enter the tumour microenvironment (TME), their phenotype and function are affected by a complex immunosuppressive network of cancer cells, inflammatory cells, suppressive cytokines and stromal cells. These components drive T-cells to differentiate into ‘exhausted’ T-cells displaying decreased effector cytokine secretion and impaired cytotoxicity causing an inability to control cancer growth [9]. T-cell exhaustion is accompanied by a progressive increase in the expression level and diversity of inhibitory receptors, including PD-1, LAG3, Tim-3, CD152 (CTLA-4) and TIGIT. In RCC, co-expression of PD-1 and Tim-3 on TILs has been associated with higher stages of the disease and a poor clinical outcome [10, 11].

However, targeted immune checkpoint blockade strategies have only been shown to be effective in a subset of patients (~ 25% of patients in the case of nivolumab), indicating that factors beyond this inhibitory axis are shaping the immune control of tumours. It is becoming increasingly apparent that the fate and function of T-cells are intrinsically tied to their metabolism, and T-cells require the machinery to fulfil their bioenergetic and biosynthetic needs to support proliferation and effector function [12]. Thus, it is unsurprising that T-cells fail in the complex TME of RCC, which can be characterized by loss of function of the tumour suppressor protein von Hippel Lindau (pVHL) resulting in aberrant activation of the hypoxia inducible factors HIF-1*α* and HIF-2*α* [13]. The resulting pseudohypoxic phenotype in RCC tumours leads to massive angiogenesis and dysregulated metabolism of the tumour cells themselves limiting nutrients and accumulating immunosuppressive waste products [14]. Thus, the complex signals within the TME promotes effector T-cells with metabolic needs that cannot be met resulting in a loss of tumour immunity.

Recently, Siska PJ et al. [15] investigated the functionality and intrinsic metabolism of ccRCC TILs from a clinically undefined cohort of patients with RCC and found them to be phenotypically distinct and both functionally and metabolically impaired, with the TILs unable to efficiently uptake glucose or perform glycolysis and demonstrating elevated mitochondrial ROS.

Here, we also investigated the phenotype, function and metabolic capability of TILs from RCC but correlated this with the clinicopathological features of the tumour and the metabolic TME comparing pT1-2 to the normal baseline control (normal kidney), pT3-4 to stage pT1-2 and thrombi and RCCmet to stage pT3-4. In addition, we explored whether the observed emergence of T-cell dysfunction in ccRCC at advanced stages of disease is associated with metabolic alterations within the tumour microenvironment. Our results demonstrate that changes in the metabolic landscape of ccRCC result in a ‘tipping-point’ during disease progression promoting immune dysfunction, cancer progression and poor outcomes from this disease.

## Materials and methods

### Human tissue

The study evaluated tumour tissue and blood samples from renal cancer patients from the Royal Marsden Hospital (London, UK) and Frimley Park Hospital (Camberley, UK). Written informed consent was obtained and the donation of tumour tissue and blood samples for evaluation had received Research Ethics Committee approval (study no: 12/L0/1661).

### Isolation of human peripheral blood mononuclear cells

Whole blood collected in BD Vacutainer blood tubes (BD Biosciences, UK) was diluted 1:1 in RPMI1640 medium and separated by centrifugation on Histopaque. Peripheral blood mononuclear cells (PBMCs) were harvested from the interface, washed and counted.

### Dissociation of human tumour tissue

RCC tumour biopsy samples, surplus to diagnostic requirements, were collected immediately following surgical resection and transported cold directly to the laboratory for processing. Tumour biopsies were subjected to a commercial mechanical/enzymatic dissociation system (GentleMACS, Miltenyi Biotec, Germany). After disaggregation, the TILs suspension was passed through 70-μm strainers and treated with red blood cell lysis solution (Qiagen). Following washing, the TILs were resuspended in RPMI1640/10%FCS and counted using a haemocytometer.

### Flow cytometry

Analysis of TILs from RCC tumour and blood samples was performed on cells directly following isolation. The following anti-human antibodies were used: CD45-V450, CD4-APC or CD8-APC, CCR7-PE, CD27-APCVio770 (Miltenyi Biotec), PD-1-PECy7 (BD Biosciences), LAG-3-FITC (R&D Systems), TIGIT-FITC (eBioscience), anti-TIM3- PerCPCy5.5 (eBioscience), BTLA-PerCPCy5.5 (BioLegend) and CD39-PerCyPCy5.5 (BD Biosciences). Cells were stained in PBS/1% FCS (Miltenyi Biotec) for 30 min at 4 °C. JC-1 (2 μM) (Molecular Probes), MitoTracker Green (100nM) (Invitrogen), MitoSOX (Invitrogen) and 2-NBDG (Invitrogen) were used per manufacturer’s instructions. Acquisition was performed on a MACSQuant flow cytometer (Miltenyi Biotec), and the MACSQuantify software was used for analysis.

### Intracellular cytokine release

PBMCs were stimulated on anti-CD3 (OKT3) (eBioscience)-coated plates plus soluble anti-CD28 (eBioscience) or left unstimulated overnight. 1μg/ml brefeldin A (Sigma-Aldrich) was added for the last 4hrs of culture. Cells were then harvested and stained for surface markers CD45, CD8, CD4 (Miltenyi) and PD-1 (BD Biosciences). Cells were fixed and permeabilized for detection of intracellular molecules using anti-IFNγ and TNF*α* (Miltenyi Biotec). Samples were acquired on a MACSQuant flow cytometer (Miltenyi Biotec), and the MACSQuantify software was used for analysis.

### Seahorse metabolic assay

The oxygen consumption rate (OCR) and extracellular acidification rate (ECAR) were measured on an XF Extracellular Flux Analyser (Seahorse Bioscience). CD3 T-cells were stimulated with 1 μg/ml CD3 (OKT3) (eBioscience) and 20U/ml IL-2 in non-buffered RPMI1640 medium during the assay. Inhibitors were 1 μM oligomycin, 1.5 μM FCCP, 1 μM antimycin A (Sigma-Aldrich) and 100 nM rotenone (Seahorse Bioscience).

### Total RNA extraction from FFPE-tumour tissues

Total RNA was isolated from paraffin-embedded tumour tissues using Norgen FFPE RNA Purification kit (Norgen Biotek, Canada) as per the manufacturer's instructions. The RNA concentration and purity was measured using an Agilent 2100 Bioanalyser.

### Nanostring

Digital multiplexed NanoString nCounter analysis system (NanoString Technologies, USA)-based gene expression profiling was performed on 100 ng total RNA from each sample according to the manufacturer’s instructions. Nanostring RNA analysis of 180 metabolism-related human genes was performed using the nCounter GX Human Cancer Metabolism profiling Kit (XT) on the nCounter® Analysis System. Analysis and normalization of the raw Nanostring data were performed using nSolver Analysis Software v1.1 (Nanostring Technologies).

### Analysis of the associations between gene expression, overall survival and clinicopathology

TCGA gene expression quantification for ccRCC (TCGA-KIRC) was downloaded from the TCGA repository (https://portal.gdc.cancer.gov/repository, accessed: 19 May 2019) consisting of RNA-seq high-throughput sequencing count files. Of 538 primary solid tumour samples, 367 are stage pT1-2, 241 are stage pT3-4 and three are ‘not reported’. The edgeR bioconductor package (v3.32.1) was used for data pre-processing {https://doi.org/10.1093/bioinformatics/btp616}. Lowly expressed genes were filtered out by keeping genes with Counts Per Million (CPM) >  0.129 (median CPM of 8 counts) in at least 25% of samples. Data were normalized using the trimmed mean of M-values normalization method (TMM). Survival analyses were performed using R packages (Rv4.0.4) survival (v3.2–7) and survminer (v0.4.9) on TMM normalized log2(CPM). Each gene was assessed through a univariate Cox regression model and overall survival Hazard ratios (with 95% confidence interval) calculated using stratified gene expression (high = above 75%, low = below 25%). Kaplan–Meier overall survival analyses of patients stratified according to gene expression (high = above 75%, low = below 25%) were performed, and log rank p-values were calculated.

### Statistical analysis

Statistical analyses were performed using two-way ANOVA or paired Student t tests as appropriate, and significant differences are indicated in the figures (∗*p* < 0.05; ∗∗*p* < 0.01; ∗∗∗*p* < 0.001).

## Results

### PD1 expression on RCC TILs increases with stage of disease

A total of 41 patients were included in the study. Of these 41 patients, *n* = 32 primary tumour samples, *n* = 9 samples of locally advanced tumour extending into the lumen of the inferior vena cava, *n* = 6 distant metastases (adrenal and lymph node) and *n* = 3 benign cases were studied (Table 1).

**Table 1:** Summary of clinical characteristics of ccRCC patient samples used in this study

Clinical characteristics of ccRCC patients	
Men	*n* = 27 (65.9%)
Women	*n* = 14 (34.1%)
Age (Average in years)	
Men	56 (range: 32–80)
Women	63 (range: 39–78)
Tumour stage (primary)	
pT1a	*n* = 7 (21.9%)
pT1b	*n* = 8 (25%)
pT2a	*n* = 2 (6.3%)
pT3a	*n* = 10 (31.3%)
pT3b	*n* = 5 (15.6%)
Locally advanced disease	
IVC	*n* = 9
Distant metastasis	*n* = 6
Benign	*n* = 3

**Fig. 1:**
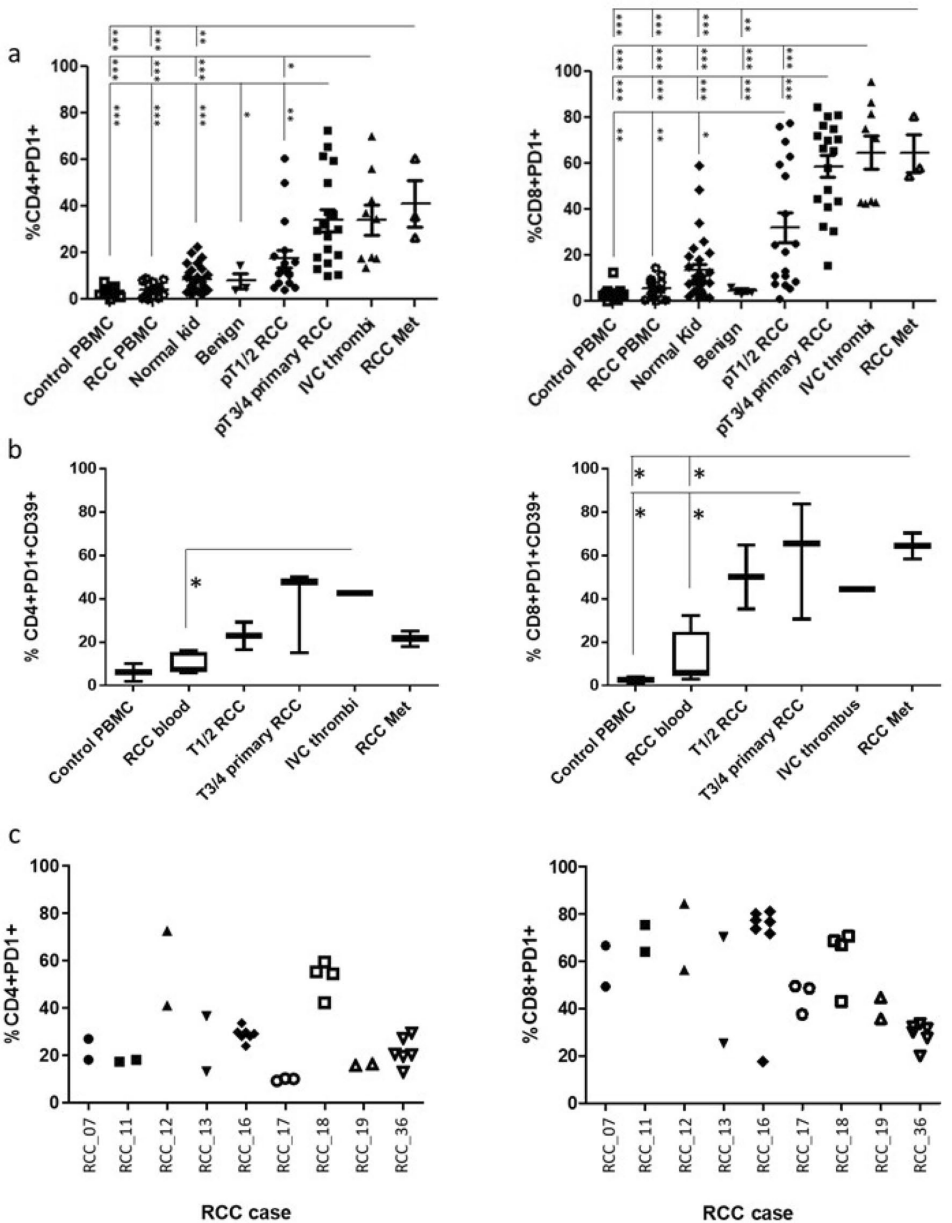
Increase in exhausted phenotype of RCC TILs in advanced stages of disease **a** Flow cytometric analysis of PD1 expression on CD4 and CD8 T-cells derived from healthy control donor bloods (*n* = 11), RCC patient bloods (*n* = 14), normal kidney tissues (*n* = 29), benign kidney tumours (*n* = 3), and RCC tumours at stage pT1-2 (*n* = 18), pT3-4 (*n* = 18), IVC thrombi (*n* = 9) and RCC metastatic (*n* = 3). **b** Multicolour flow cytometric analysis of CD4+PD1+ and CD8+PD1+ T-cells for their co-expression of CD39. **c** Flow cytometric analysis of PD1 expression on CD4 and CD8 T-cells derived from multiple biopsy sites within the same tumour from *n* = 9 advanced pT3-4 RCC tumours. Significant differences between sample types was determined by two-way ANOVA; ∗*p* < 0.05; ∗∗*p* < 0.01; ∗∗∗*p* < 0.001

Firstly, we evaluated PD-1 expression on freshly resected and dissociated RCC tumour cell suspensions from 17 stage pT1-2 tumours, 15 stage pT3-4 tumours, nine locally advanced IVC tumour thrombi and three distant RCC metastatic sites and compared them to cell suspensions derived from benign cases, normal kidney tissues, blood from RCC patients and blood from healthy controls. As shown in Fig. 1a, PD1 expression on T-cells increased with disease stage with pT3-4 RCC tumours, locally advanced tumours and metastatic sites displaying the highest expression of PD-1 on CD4+ and CD8+T-cells. By comparison, the low level of PD1 expression on T-cells was the same for all the control tissues (benign tumours, normal kidney tissue, blood from RCC patients and healthy controls). To provide more evidence that these high expressing PD-1+TILs from the more advanced stage tumours were indicative of exhausted T-cells, in some of the cases, CD39 (a marker associated with an exhausted subset of CD8^+^ T-cells [16]) was combined with PD1 and T-cell markers. CD39 expression on PD-1+TILs increased with disease stage mirroring the increase in PD-1 expression (Fig. 1b). From nine of the advanced pT3-4 RCC tumours, we obtained multiple biopsies taken from different sites within the tumour. While the expression of PD1 on T-cells isolated from different sites within a tumour was largely the same, there were several cases where there was significant heterogeneity in PD1 expression between different biopsy sites (Fig. 1c). Additional inhibitory receptors (TIM3, LAG-3, TIGIT and BTLA) were also investigated on the RCC TILs. There was no significant increase in expression of these receptors on CD4+T-cells except for TIGIT which was expressed by the majority of TILs from metastatic sites. Notably, the expression of TIM3 was increased on TILs from some of the more advanced disease stages, in particular the RCC metastatic sites (Sup Fig. 1).

### RCC TILs from advanced stage disease show reduced capability to produce Th1-cytokines upon stimulation

**Fig. 2:**
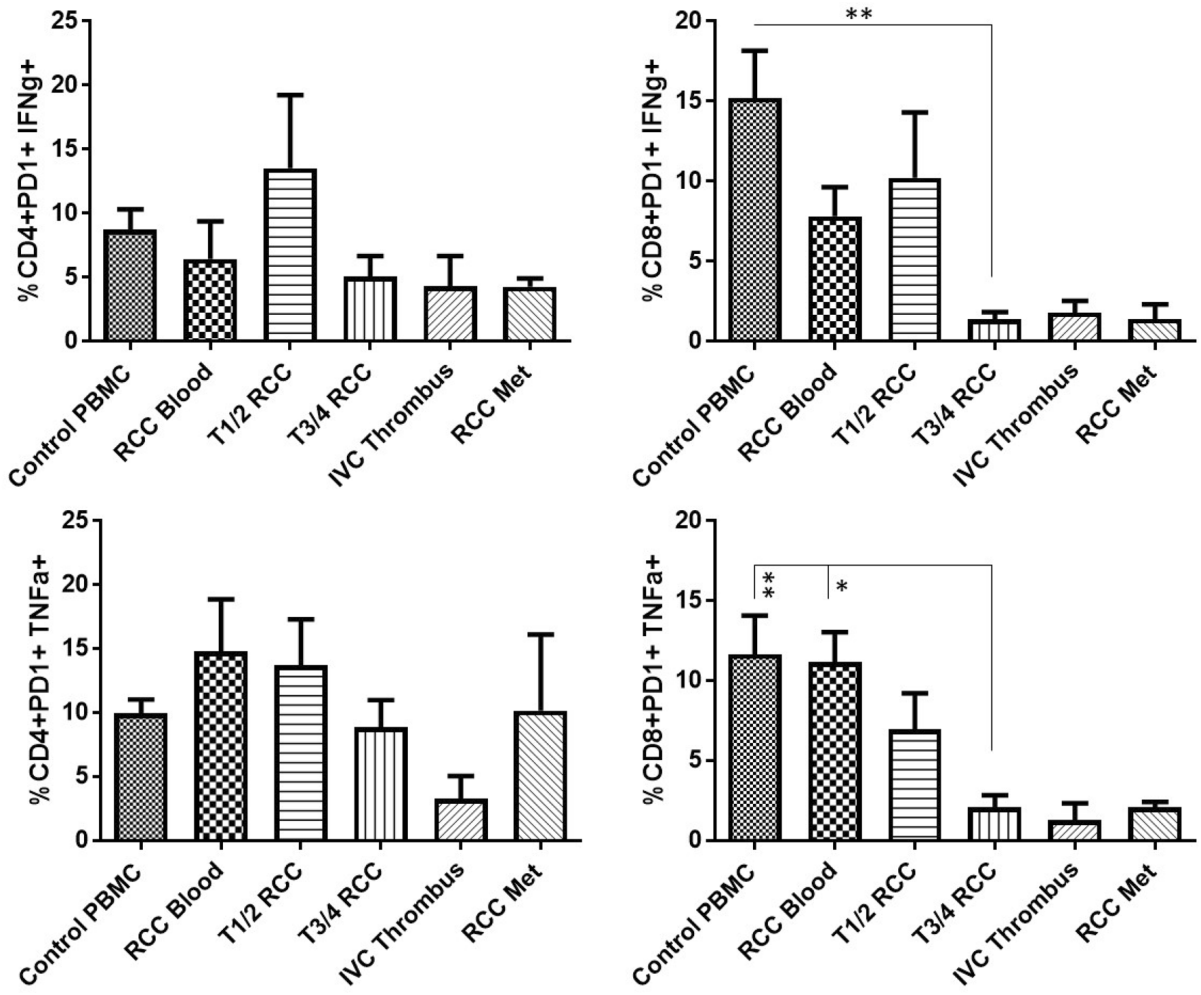
CD8+RCC TILs at advanced stages of the disease show a significantly reduced capability to produce effector cytokines upon stimulation. Freshly dissociated single-cell suspensions of RCC tumours at different stages of the disease (pT1-2 *n* = 5, pT3-4 *n* = 8, IVC thrombi *n* = 3 and RCCmet *n* = 2) along with control PBMC (*n* = 9) and RCC PBMC (*n* = 7) were stimulated *in vitro* with and without *α*CD3 and *α*CD28 and the production of effector cytokines, IFNϒ and TNF*α*, by CD4 and CD8T cells assessed by intracellular cell staining and flow cytometry. Background levels of cytokines detected within unstimulated controls were subtracted from the results obtained from stimulated samples. Significant differences between sample types were determined by two-way ANOVA; ∗*p* < 0.05; ∗∗*p* < 0.01; ∗∗∗*p* < 0.001

Single-cell suspensions of RCC tumours at different stages of the disease were stimulated *in vitro* to assess the ability of the T-cells to produce IFNϒ and TNF*α* and compared to T-cells from the blood of the patients or healthy controls. As shown in Fig. 2 the CD8+RCC TILs, from more advanced stages of the disease, showed a significantly reduced capability to produce both IFNϒ and TNF*α* upon stimulation. This contrasted with the CD8+RCC TILs from early stage (pT1-2) RCC tumours which retained their ability to produce Th1-cytokines. The CD4+RCC TILs followed a similar trend to the CD8+T-cells but with a lesser impairment to cytokine production in advanced stage disease.

### Impaired metabolic function of RCC TILs in advanced stage disease

**Fig. 3:**
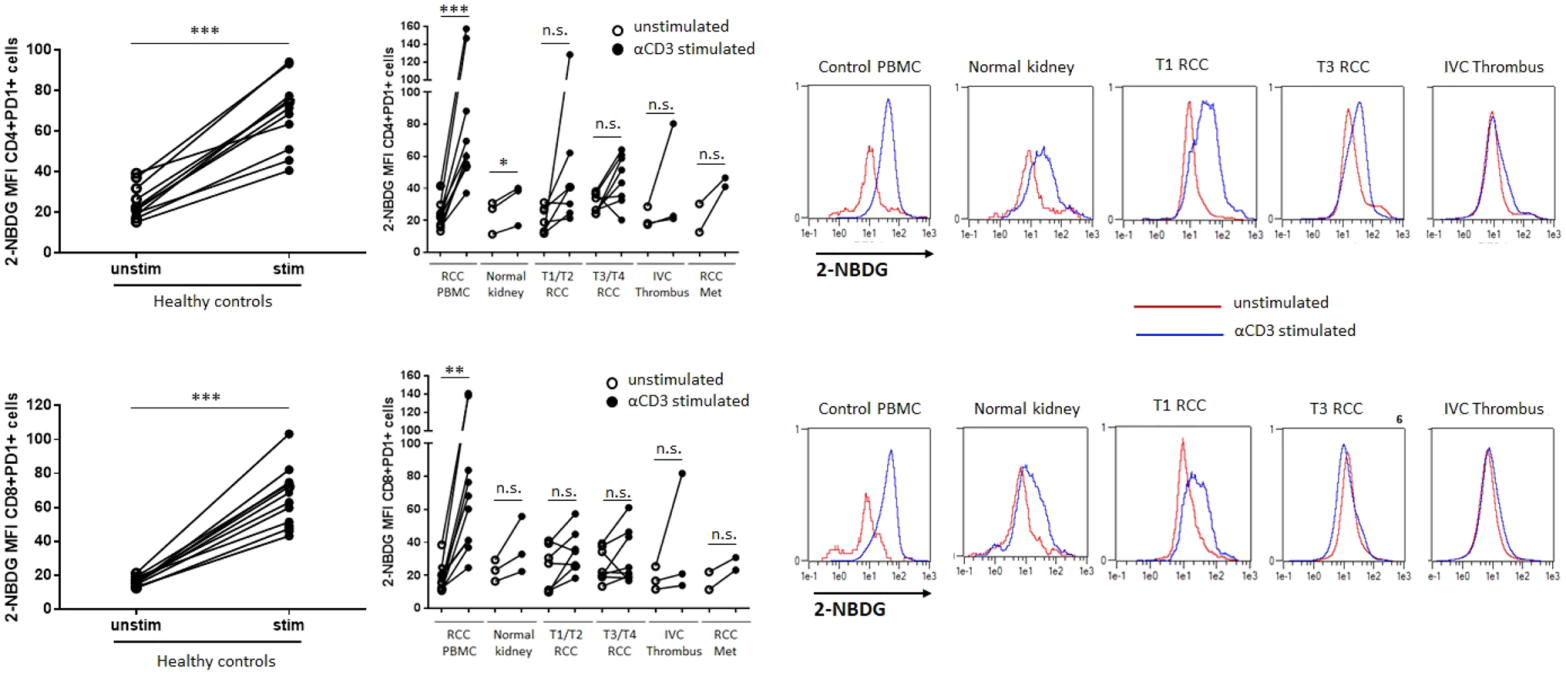
RCC TILs show decreased ability for glucose uptake freshly isolated PBMC from healthy donors (*n* = 11) or RCC patients (*n* = 5) or cell suspensions from dissociated normal kidney (*n* = 3) or RCC tumour tissues at different disease stages (pT1-2 *n* = 5, pT3-4 *n* = 4, IVC thrombi *n* = 3, RCCmet *n* = 2) was stimulated with or without plate-bound *α*CD3 for 48 h. The cells then underwent a short-time incubation in a glucose-free media (to normalize the glucose uptake rate across groups) followed by a 20–30-min incubation with 2NBDG. The cells were washed, stained for cell surface markers and the 2NBDG measured in the FL-1 (FITC) channel as a fluorescent indicator for direct glucose uptake measurement. For comparison between unstimulated and stimulated groups, paired Student *t* tests were used. Representative histogram plots are shown for CD4+PD1+ and CD8+PD1+ T-cells from PBMC and RCC tumours at different stages of disease

To understand the functional impairment of RCC TILs during advanced stages of the disease, various assays to measure the metabolic reprogramming of the T-cells, required for effector function, were utilized. First, to examine the capacity for glucose uptake by RCC TILs, we pulsed T-cells with the fluorescent glucose analogue 2-NBDG at the end of culture with or without TCR (T-cell receptor) stimulation. While the basal uptake of 2-NBDG by resting PD1+RCC TILs was similar to resting control T-cells, following stimulation RCC TILs had defects in glucose uptake. This was evident across all stages of RCC but more evident from stage pT3-4 and from the locally advanced and metastatic sites (IVC). This contrasted with the T-cells from the blood of RCC patients that were able to increase glucose uptake upon stimulation (Fig. 3).

**Fig. 4:**
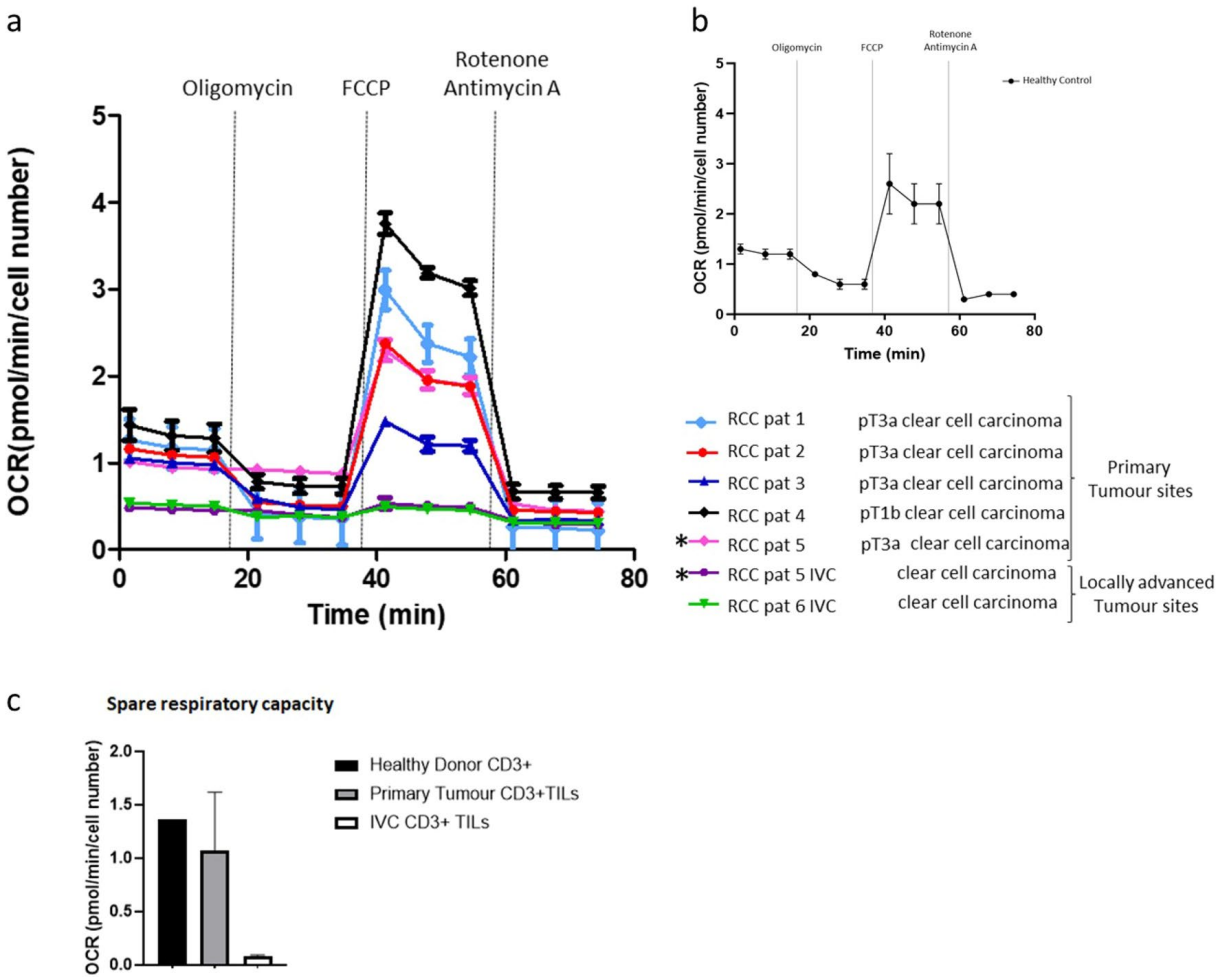
Low spare respiratory capacity of RCC TILs derived from invasive tumour sites. The metabolic phenotype of isolated RCC-derived CD3+ TILs **a** and healthy donor isolated CD3+ cells **b** was measured by assessing the cellular oxygen consumption rates during a mitochondrial stress test. **c** The spare respiratory capacity (SRC) was calculated as change in mean OCR upon treatment with FCCP (fluorocarbonyl cyanide phenylhydrazone) from basal respiration of CD3+ TILs from RCC primary tumour sites (*n* = 5) vs. locally advanced tumours exhibiting invasive morphology (IVC *n* = 2)) and healthy control CD3+T-cells (*n* = 3) as a control. The * indicates TILs derived from the primary tumour site and IVC thrombus site from the same patient (RCC 5)

To further probe the metabolic phenotype of RCC-derived TILs, we measured cellular oxygen consumption rates (OCRs) during a mitochondrial stress test. The spare respiratory capacity (SRC), calculated as change in mean oxygen consumption rate upon treatment with FCCP (fluorocarbonyl cyanide phenylhydrazone), of sorted CD3+TILs from RCC tumours varied. Most stage pT1-2 RCC tumours displayed TILs with a high SRC, while the TILs from tumours exhibiting invasive morphology (>= pT3/IVC thrombus sites) often had a low/minimal SRC. This inability to generate additional energy through oxygen consumption in situations of metabolic stress was specific to TILs from advanced stage cases and not observed in the early stage tumours or corresponding blood of RCC patients (Fig. 4).

**Fig. 5:**
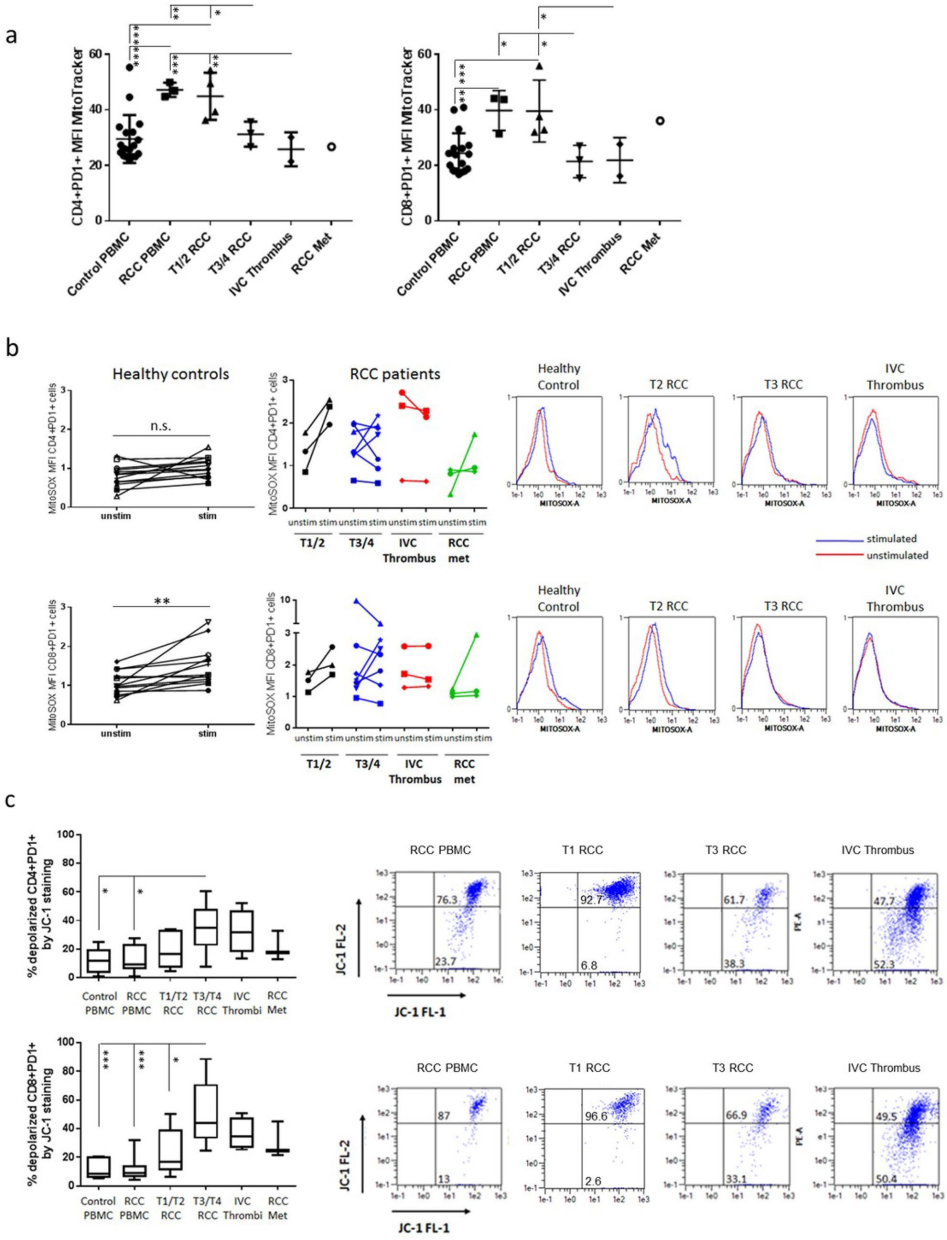
Dysfunctional depolarized mitochondria within advanced RCC TILs. **a** Freshly isolated PBMC from healthy controls (*n* = 16) or RCC patients (*n* = 3) and TILs derived from RCC tumours at different disease stages (pT1-2 *n* = 4, pT3-4 *n* = 3, IVC thrombi *n* = 2, RCCmet *n* = 1) were stimulated overnight with plate-bound *α*CD3 and 5 ng/ml IL-2. The cells were then stained with surface antibodies to identify CD4+PD1+ and CD8+PD1+ T-cells. Cells were then incubated with the MitoTracker^®^ Green dye and median fluorescence intensity (MFI) of the MitoTracker^®^ Green dye in CD4+ and CD8+ PD1+ T-cells measured by flow cytometry. **b** Mitochondrial superoxide content was detected in unstimulated and anti-CD3 stimulated TILs from >= pT3 tumours (pT3-4 *n* = 7, IVC thrombi *n* = 3, RCCmet *n* = 3) as compared to TILs from pT1-2 RCC (*n* = 3) or the T-cells from the blood of healthy controls (*n* = 13). Representative histogram plots are shown for unstimulated and anti-CD3 stimulated CD4+PD1+ and CD8+PD1+ T-cells from PBMC and from tumours at different stages of disease. **c** The fluorescent dye JC-1 was used to assess mitochondrial potential, a key readout of mitochondrial function. Following an overnight incubation with plate-bound anti-CD3 and 5 ng/ml IL-2 stimulation, mitochondrial depolarization of TILs from RCC tumours at different disease stages (pT1-2 *n* = 5, pT3-4 *n* = 10, IVC thrombi *n* = 4, RCCmet *n* = 3) and T-cells from the blood of healthy controls (*n* = 6) and RCC patients (*n* = 11) was indicated by a decrease in the red/green fluorescence intensity ratio. Representative FACS plots are shown for CD4+PD1+ and CD8+PD1+ T-cells from PBMC and from tumours at different stages of disease. Significant differences between sample types were determined by two-way ANOVA; ∗*p* < 0.05; ∗∗*p* < 0.01; ∗∗∗*p* < 0.001

As depleted mitochondrial mass may be one cause of decreased respiration, MitoTracker Green (MTG) staining of RCC-derived PD1+TILs was carried out. This analysis revealed that while the TILs from early stage (pT1-2) RCC cases had an equivalent mass to T-cells from the blood of RCC patients, the TILs from advanced stage disease showed a decreased mitochondrial mass (Fig. 5a).

We next investigated mitochondrial function by analysing the production of mitochondrial ROS levels in TILs from RCC patients using the mt-superoxide-specific dye MitoSOX Red and compared them with those of T-cells from the blood of the patients and healthy controls. The mitochondrial superoxide content was similar in unstimulated TILs from all stages of RCC as compared to the T-cells from the blood of patients or healthy controls except for two cases of IVC thrombus and a stage 3 tumour which displayed very high levels of basal ROS. Upon anti-CD3 stimulation, superoxide levels consistently increased in T-cells from the blood of control donors and from TILs from stage pT1-2 RCC patients, whereas a mixed response with most samples exhibiting a decline or no change of superoxide levels was observed in TILs from stage pT3-4 RCC patients and from IVC thrombus and metastatic sites (Fig. 5b).

Further investigation of the mitochondrial function of RCC TILs was carried out by examining the mitochondrial membrane potential (MMP) in freshly isolated TILs from different stages of RCC, after overnight stimulation with anti-CD3 antibody, using the lipophilic cation JC-1. JC-1 is mitochondria selective and forms aggregates in polarized mitochondria that result in a green-orange emission after excitation. However, the monomeric form present in cells with depolarized mitochondrial membranes emits only green fluorescence. After anti-CD3 stimulation, TILs from stage pT3-4 RCC patients and from locally advanced (IVC thrombus) and metastatic sites displayed an increased percentage of cells with mitochondrial depolarization as compared to TILs from stage pT1-2 RCC patients or T-cells from the peripheral blood of patients or healthy controls (Fig. 5c). This decreased MMP together with the inability to increase ROS levels upon stimulation further supports the existence of dysfunctional depolarized mitochondria within advanced RCC TILs.

### Metabolic profiling of the RCC tumour microenvironment reveals metabolic reprogramming from an early stage of disease

**Fig. 6:**
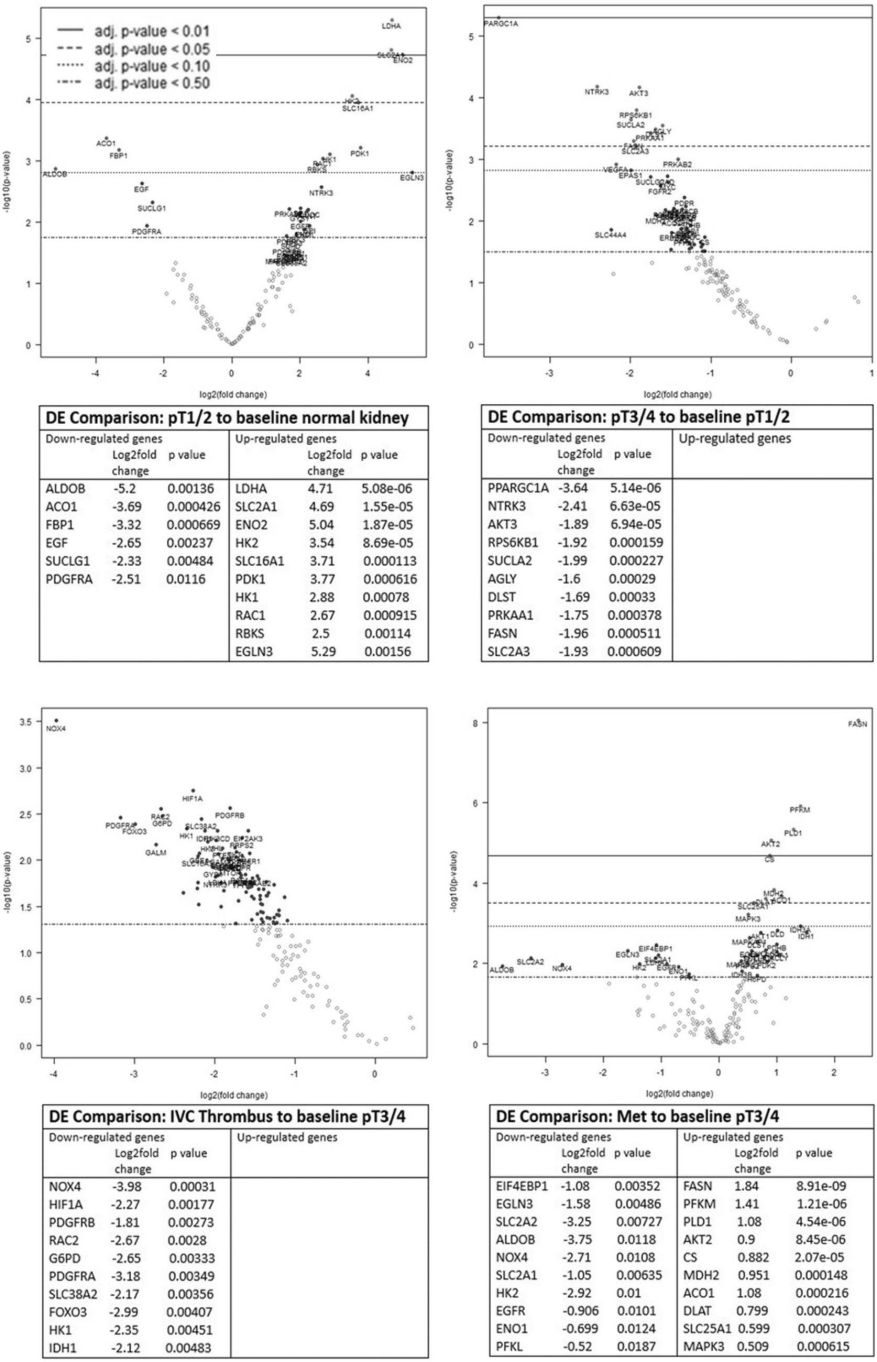
Differential metabolism gene expression analysis during RCC disease progression. Nanostring nCounter cancer metabolism panel assay was performed on RNA obtained from 30 ccRCC cases. Differential gene expression analysis was determined for each stage of the disease (pT1-2 *n* = 16), p3-4 (*n* = 10), IVC thrombi *n* = 5 and RCCmet *n* = 5) comparing pT1-2 to the normal baseline control (normal kidney), pT3-4 to stage pT1-2 and thrombi and RCCmet to stage pT3-4. For each of these disease stage comparisons, Volcano plots display each gene’s −log10(*p* value) and log2fold change with the highly statistically significant genes labelled and falling at the top of the plot above the horizontal lines (*p* value thresholds). The tables list the top 10 up- and down-regulated differentially expressed metabolism genes for each disease stage comparison

To explore whether the observed emergence of T-cell dysfunction in ccRCC at advanced stages of disease is associated with metabolic alterations within the tumour microenvironment, a Nanostring nCounter cancer metabolism panel assay was performed on RNA obtained from 30 of the ccRCC cases studied above. Differential gene expression (DGE) analysis was determined for all ccRCC cases compared to normal kidney (Fig. 6) and for each stage of the disease (pT1-2, pT3-4, IVC thrombi and RCCmet) comparing pT1-2 to the normal baseline control (normal kidney), pT3-4 to stage pT1-2 and thrombi and RCCmet to stage pT3-4. (Fig. 6). The DGE analysis of all ccRCC cases compared to normal kidney tissue (Sup Fig. 2) was largely reflective of the up- or down-regulated genes revealed at pT1-2 stage of the disease (Fig. 6A). Even during these early stages of the disease (pT1-2), there was over-expression of genes (*LDHA*, *SLC2A1*, *HK2*, *SLC16A1*, *HK1*) corresponding to proteins associated with glucose metabolism and solute transport functions that could potentially render the cancer cells more fit while at the same time imposing metabolic effects (nutrient deficiency and waste product toxicity) that restrict the TILs. In addition, *PDK1*, a gene known to regulate RCC cell proliferation, migration, invasion and epithelial mesenchymal transition, as well as HIF-responsive genes, *ENO2* and *EGLN3*, were also over-expressed.

Similarly, we observed down-regulation of particular metabolic genes (ALDOB, AC01, FBP1, EGF, SUCLG1 and PDGFRA) also from an early (pT1-2) stage of disease. These findings were in keeping with previous reports showing that *ALDOB* and *FBP1* were some of the most down-regulated genes in RCC [17, 18].

Progression into pT3-4 stage disease compared to pT1-2 and thrombi compared to pT3-4 showed an overall down-regulation of metabolic genes (Fig. 6b). Of note, there was a significant down-regulation of *PPARGC1A* in pT3-4 stage disease, a gene that encodes peroxisome proliferator-activated receptor gamma coactivator-1, consistent with previous reports of low expression of *PPARGC1A* in ccRCC tissues [19]. In thrombi compared to pT3-4 stage disease, there was a significant down-regulation of the kidney-specific NADPH oxidase isoform 4 (*NOX4*) which produces considerable amounts of ROS in ccRCC. In the most advanced stage of the disease, the RCCmets compared to pT3-4 stage showed an up-regulation of genes (*FASN, PFKM, PLD1, AKT2, Citrate synthase (CS) and MDH2*) that are associated with aggressive cell proliferation, migration, apoptosis, lipid droplet formation and regulate metabolic disorders of the ccRCC microenvironment. Similar to the thrombi, RCCmet showed a significant down-regulation of *NOX4* along with aldolase B (*ALDOB*) and the glucose solute carrier, *SLC2A2*.

**Fig. 7:**
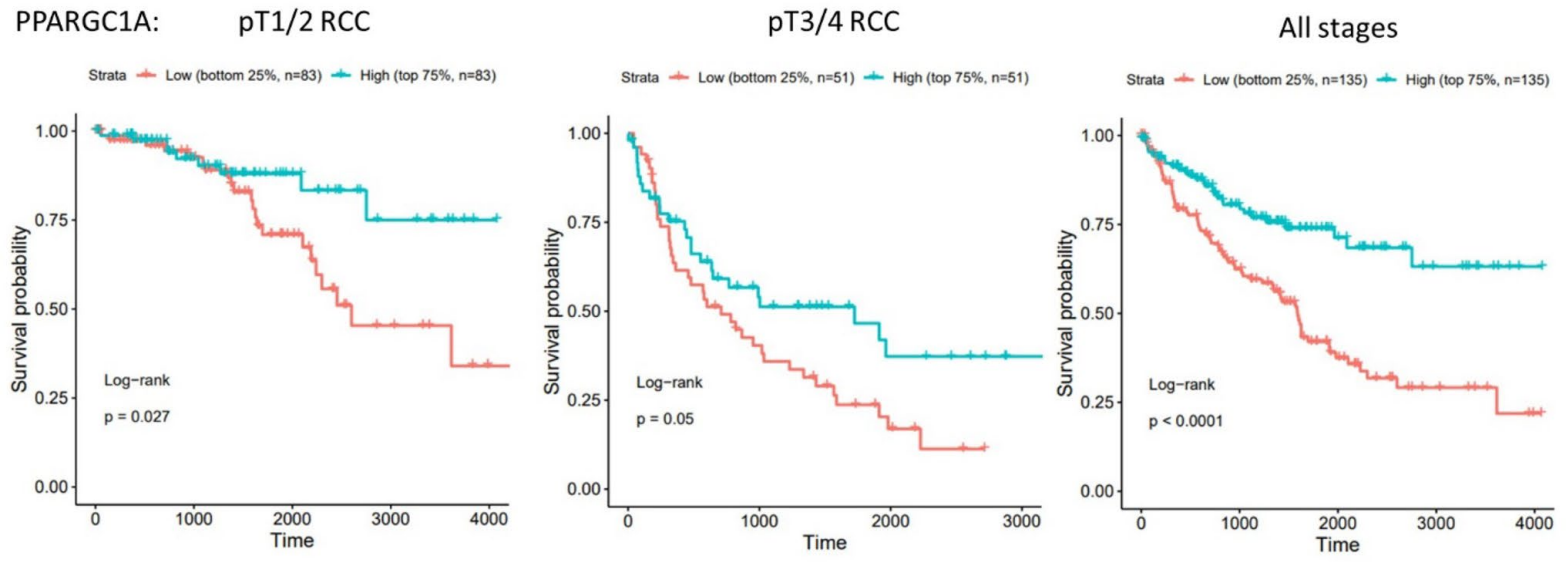
*PPARGC1A* expression is associated with prognosis in clear cell renal cell carcinoma. Kaplan–Meier overall survival of patients stratified according to *PPARGC1A* gene expression as high (above 75%) and low (below 25%), in the TCGA-KIRC dataset, at different stages of renal cell carcinoma (left: stage pT1-2, centre: stage pT3-4 and right: all stages)

The most significantly up- or down-regulated genes from each stage comparison were then selected for analysis of associations with overall survival (OS) using mRNA expression data from the TCGA database (Sup Fig. 3a–d). For genes differentially expressed in stage pT1-2 relative to normal tissue, the results demonstrated that for the up-regulated genes, increased mRNA expression level of *PDK1* was associated with improved OS, whereas the increased expression of ENO2 was linked to poor OS (Sup Fig. 3a). For the down-regulated genes *SUCLG1, FBP1, AC01* and *ALDOB*, low expression was associated with a poor OS (Sup Fig. 3a). For the majority of the genes down-regulated at stage pT3-4 (*PRKAA1, DLST, PPARGC1A, SUCLA2* and *AKT3*), low expression was significantly associated with a poor OS, and only for *SLC2A3* and *FASN,* low expression was linked with an improved OS (Sup Fig. 3b). As published work had shown that *PPARGC1A* was involved in shifting the TME from immune-dominant to metabolic-dominant [20], we explored further the association of *PPARGC1A* expression and survival within different disease stages (Fig. 7). TCGA data revealed that low expression of this gene is associated with poor survival in all stages of RCC with our own data showing a significant down-regulation of this gene in stage pT3-4 tumours compared to stage pT1-2 (Fig. 6b).

For genes down-regulated in the thrombi relative to stage pT3-4, low expression of *FOXO3* was associated with a poor outcome, whereas low expression of *G6PD* and *RAC2* was linked to improved OS (Sup Fig. 3c). For genes differentially expressed in advanced metastatic stage, relative to stage pT3-4, high expression of many of the up-regulated genes was associated with improved OS; however, high expression of *FASN* was linked to a poor outcome. Low expression of down-regulated genes *SLC2A2* and *ALDOB* was associated with a poor OS (Sup Fig. 3d).

## Discussion

RCC is a disease characterized by a metabolic signature reflecting its adaptation to hypoxia and bioenergetic needs to sustain cellular proliferation [21]. Growing evidence shows that this metabolic profile is responsible for the dysfunctional immune response of TILs which are abundant in ccRCC [14]. This ‘inflamed’ phenotype of tumour should be responsive to immunotherapies such as immune checkpoint inhibition, yet many patients still do not respond. Ascierto et al. [22] explored the gene expression landscape of PD-L1+RCCs derived from patients with divergent clinical outcomes after anti-PD-1 therapy and showed that a signature of up-regulated metabolic genes was associated with treatment failure in patients with PD-L1+RCC. Conversely, tumours from responding patients had an up-regulated immune gene signature. These findings strongly suggested that the tumour-imposed metabolic effects are responsible for the dysregulated immune response in RCC. The current study set out to confirm the dysregulated immune response in ccRCC, the stage of disease at which T-cells become affected, and any association with tumour stage-specific metabolic gene expression. Here, we show the emergence of exhausted T-cells in more advanced stages (pT3-4, IVC thrombus and RCC metastases) of the disease based on their PD-1^high^ and CD39 expression and their reduced ability to produce inflammatory cytokines upon *in vitro* stimulation. These exhausted T-cells from advanced stage disease also displayed an overall phenotype of metabolic insufficiency, characterized by mitochondrial alterations and defects in glucose uptake. Since historically the majority of RCC patients receiving immunotherapy will have had more advanced disease, the inability of their TILs to respond to immunotherapies is unsurprising given their exhausted and metabolically impaired phenotype.

To create a more permissive environment for T-cell anti-tumour activity, it is important to understand the TME factors that are negatively impacting the TILs. Given the wealth of data demonstrating the extensive metabolic reprogramming in ccRCC [23, 24], this study explored the metabolic gene expression profile at different stages of the disease that may explain the emergence of the observed exhausted tumour-infiltrating T-cells. Even though T-cells derived from early stage tumours had shown no features of exhaustion (PD-1^lo^ and CD39^lo^ and functionally produced inflammatory cytokines upon stimulation), pT1-2 tumours already had significant over-expression of particular metabolic genes. These included genes involved with solute transport functions such as *SLC2A1* which encodes the glucose transporter 1 (GLUT1) [25] and *SLC16A1* which encodes a proton-linked monocarboxylate transporter that catalyses the movement of many monocarboxylates, such as lactate and pyruvate, across the plasma membrane [26]. Genes encoding glycolytic enzymes such as *HK1*, *HK2* and *LDHA* were also over-expressed. The hexokinases phosphorylate glucose to produce glucose-6-phosphate (G6P) is the first step in most glucose metabolism pathways [27], while *LDHA* is necessary for conversion of pyruvate to lactate [28]. Additionally, HIF-responsive genes *ENO2* and *EGLN3* were among the highest expressed metabolic genes along with genes such as *PDK1* and *RAC1* which have been shown to be involved in regulating cell proliferation, migration, invasion and metastasis [29–31]. Besides over-expressed metabolic genes, several down-regulated metabolic genes (*AC01, ALDOB, SUCLG1 and FBP1*) were identified already in stage pT1-2 compared to normal tissue, whose low expression was associated with a poor OS. Indeed, *ALDOB* and *FBP1* are known to be two of the most down-regulated genes in RCC, and both are involved in gluconeogenesis [17, 18]. Fructose-1,6-bisphosphatase 1 (*FBP1*) inhibits ccRCC progression through two distinct mechanisms: (1) by antagonizing glycolytic flux in renal tubular epithelial cells, thereby inhibiting a potential Warburg effect and (2) by restraining cell proliferation, glycolysis and the pentose phosphate pathway in a catalytic-activity-independent manner, by inhibiting nuclear HIF function via direct interaction with the HIF inhibitory domain. This unique dual function of the FBP1 protein explains its ubiquitous loss in ccRCC [18]. Thus, even at early stage disease, ccRCC cells are establishing an advantage for themselves in outcompeting the TILs for vital nutrients. Furthermore, it has been shown that LDHA-associated lactic acid production can suppress T-cell and NK-cell activation and function [32]. Despite the high expression of these metabolic genes during early stage disease, their resulting restriction of nutrients and/or effect of metabolic waste products within the TME appears to take time to impact the TILs which only displayed exhaustion at advanced disease stages.

Interestingly, compared to the early disease stage pT1-2, at pT3-4 and the locally advanced thrombi stages, there was an overall decrease in differentially expressed metabolic genes. Notably, the gene *PPARGC1A* (also known as PGC1α) was significantly down-regulated in our cohort of pT3-4 stage RCC, consistent with published data [33]. *PPARGC1A* is known to be a key transcriptional coactivator that coordinates mitochondrial biogenesis and oxidative phosphorylation in tumour cells to induce metastasis [34]. Recently, Ma et al. [20] observed negative correlations between *PPARGC1A* expression and tumour grade, clinical stage, and M stage in patients with ccRCC. They showed that in high expressing *PPARGC1A* ccRCCs, immune-related signalling and epithelial mesenchymal transition pathways were the most enriched, while in low expressing tumours, metabolic pathways were highly enriched. This led the authors to speculate that *PPARGC1A* is involved in shifting the TME from immune-dominant to metabolic-dominant. Furthermore, they showed that *PPARGC1A* was negatively correlated with abundances of Tregs and CD8+T-cells. PGC1α is particularly important for TILs to maintain high mitochondrial activity which promotes CD8+T-cell fitness, memory formation and anti-tumour immunity [35]. Our data and others [36, 37] have clearly shown that tumour-infiltrating T-cells display an overall phenotype of metabolic insufficiency due to loss of mitochondrial function and mass. This study shows this loss of mitochondrial function in tumour-infiltrating T-cells is most evident at tumour stage pT3-4 which correlated with the down-regulated expression of *PPARGC1A* in ccRCC tumours at this stage. The down-regulation of *PPARGC1A* with the transition of disease stage to stage pT3-4 may be the ‘tipping-point’ in RCC disease progression. This may then modulate immune activity in the TME, and potentially reduce the efficacy of immunotherapies resulting in worse patient outcomes. While no specific drugs activating PGC1α are currently available, strategies to direct the metabolic reprogramming of T-cells such as enforcing expression of PGC1α [36] or 4-1BB co-stimulation of CD8+T-cells which engages PGC1α-mediated pathways via activation of p38-MAPK [37] result in enhanced mitochondrial capacity and have been shown to improve the anti-tumour effects of adoptive cell therapy. Combining such strategies with current immunotherapy treatments may overcome the immunosuppressive metabolic landscape of the ccRCC microenvironment and increase the proportion of patients benefiting from immunotherapy both in the adjuvant setting (improving for example single-agent pembrolizumab) and/or increase its duration of response in metastatic disease. Further insights are needed as to the effects of IO/TKI drugs on T-cell metabolism and specifically on mitochondrial function to aid clinicians as to the choice of optimal combinations which may be most likely to affect PGC1α dysregulation.

### Supplementary Information

Below is the link to the electronic supplementary material.

Supplementary file1 (DOCX 910 kb)
