# Supplementary material for: The dysfunctional immune response in renal cell carcinoma correlates with changes in the metabolic landscape of ccRCC during disease progression
Source: Cancer Immunol Immunother. 2023 Nov 8;72(12):4221–34. doi: 10.1007/s00262-023-03558-5 (PMC10700462; doi:10.1007/s00262-023-03558-5)
Supplement: Supplementary file 1 — Supplementary file1 (DOCX 910 kb) [file 262_2023_3558_MOESM1_ESM.docx]

Supplementary figures


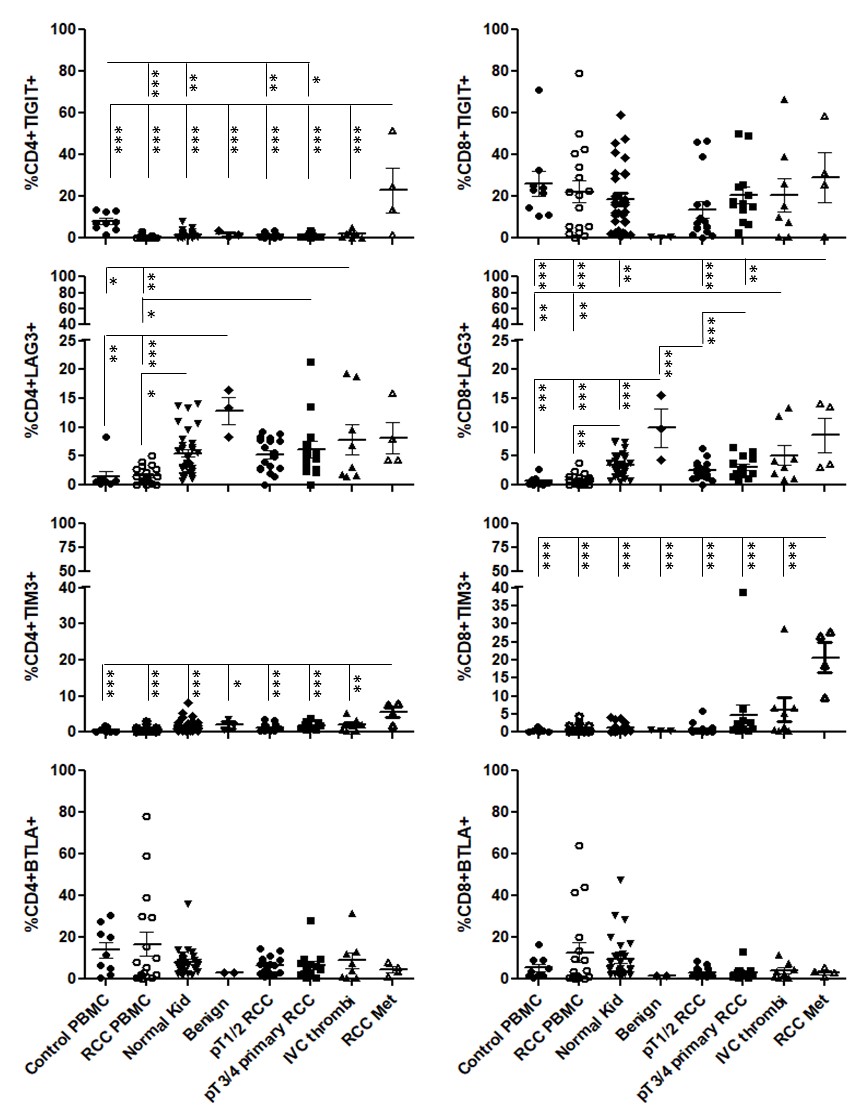


**Sup Figure 1: Inhibitory receptor expression on RCC TILs from different disease stages**

Flow cytometric analysis of TIGIT, LAG3, TIM3 and BTLA expression on CD4 and CD8 T cells derived from healthy control donor bloods, RCC patient bloods, normal kidney tissues, benign kidney tumours, and RCC tumours at stage p1/2, p3/4, IVC thrombi and RCC metastatic. Significant differences between sample types were determined by two-way ANOVA; ∗p < 0.05; ∗∗p < 0.01; ∗∗∗p < 0.001.


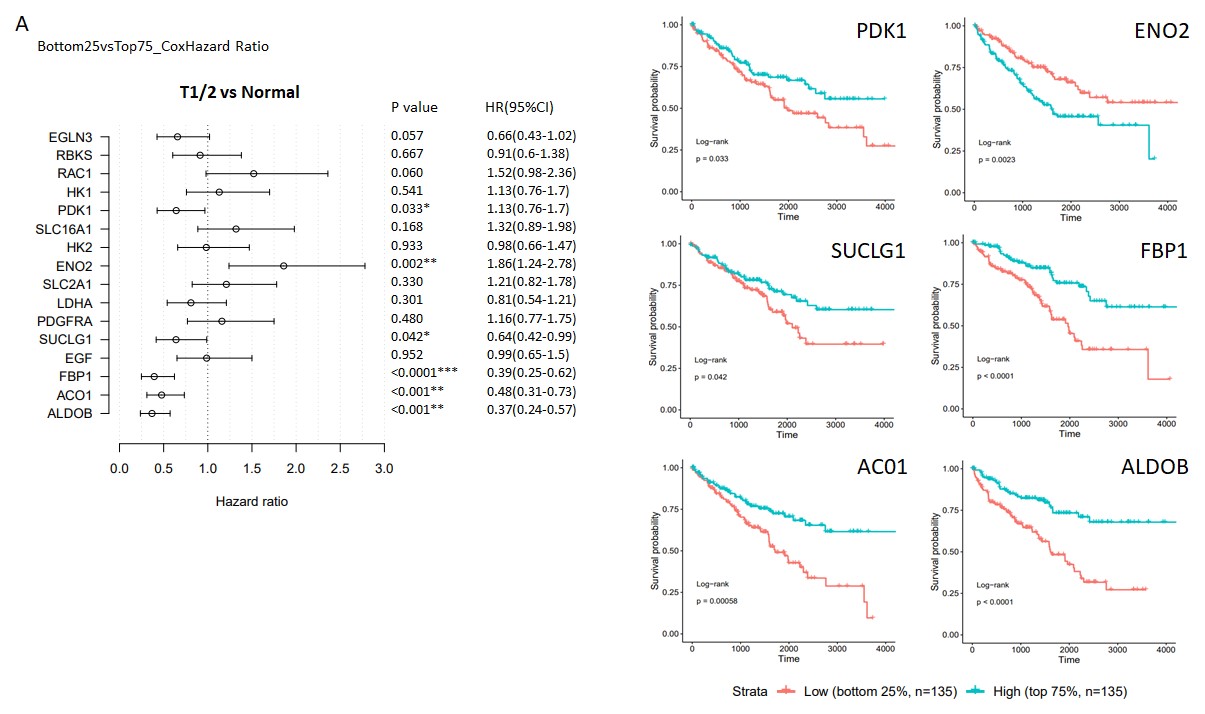


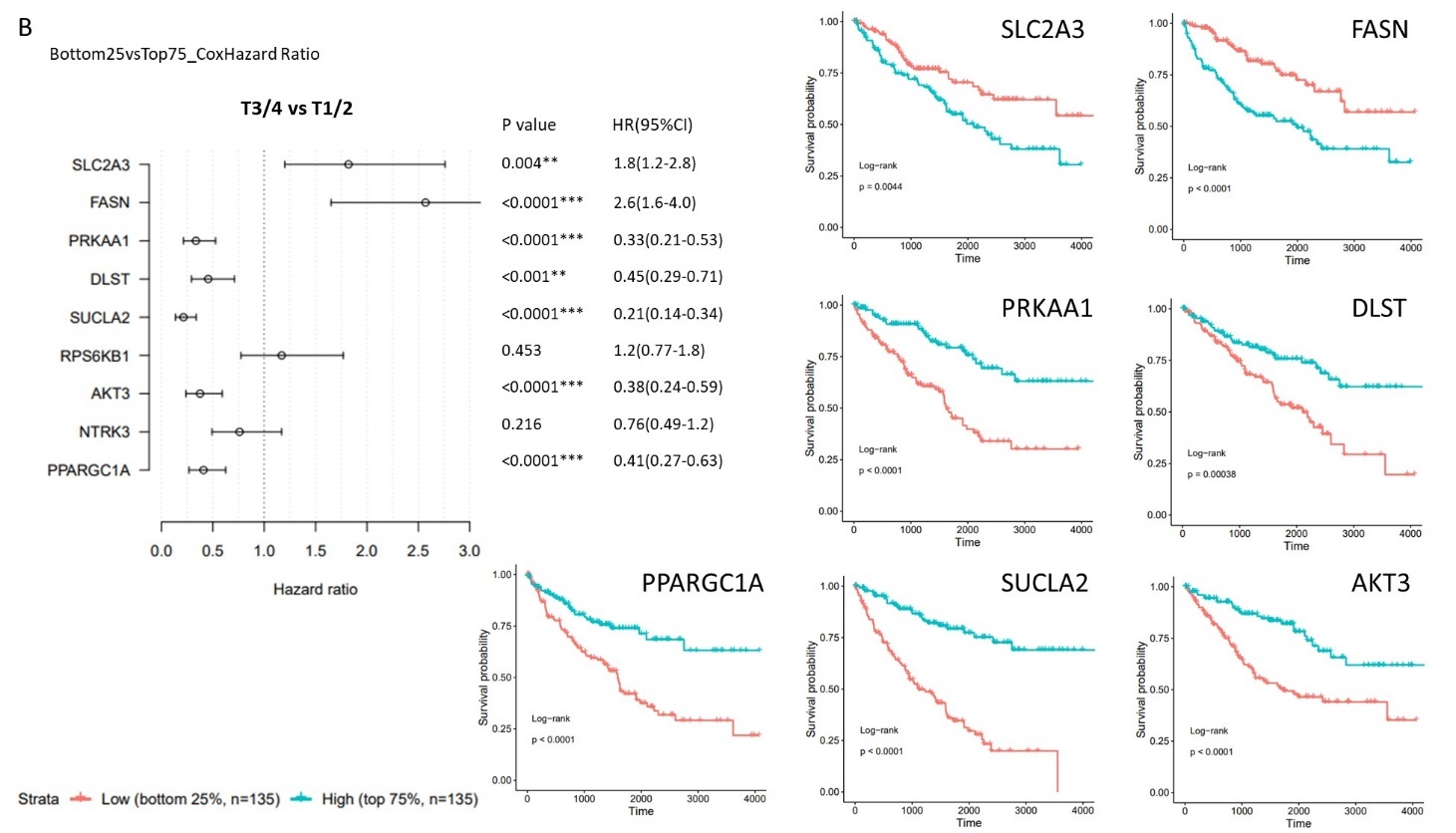


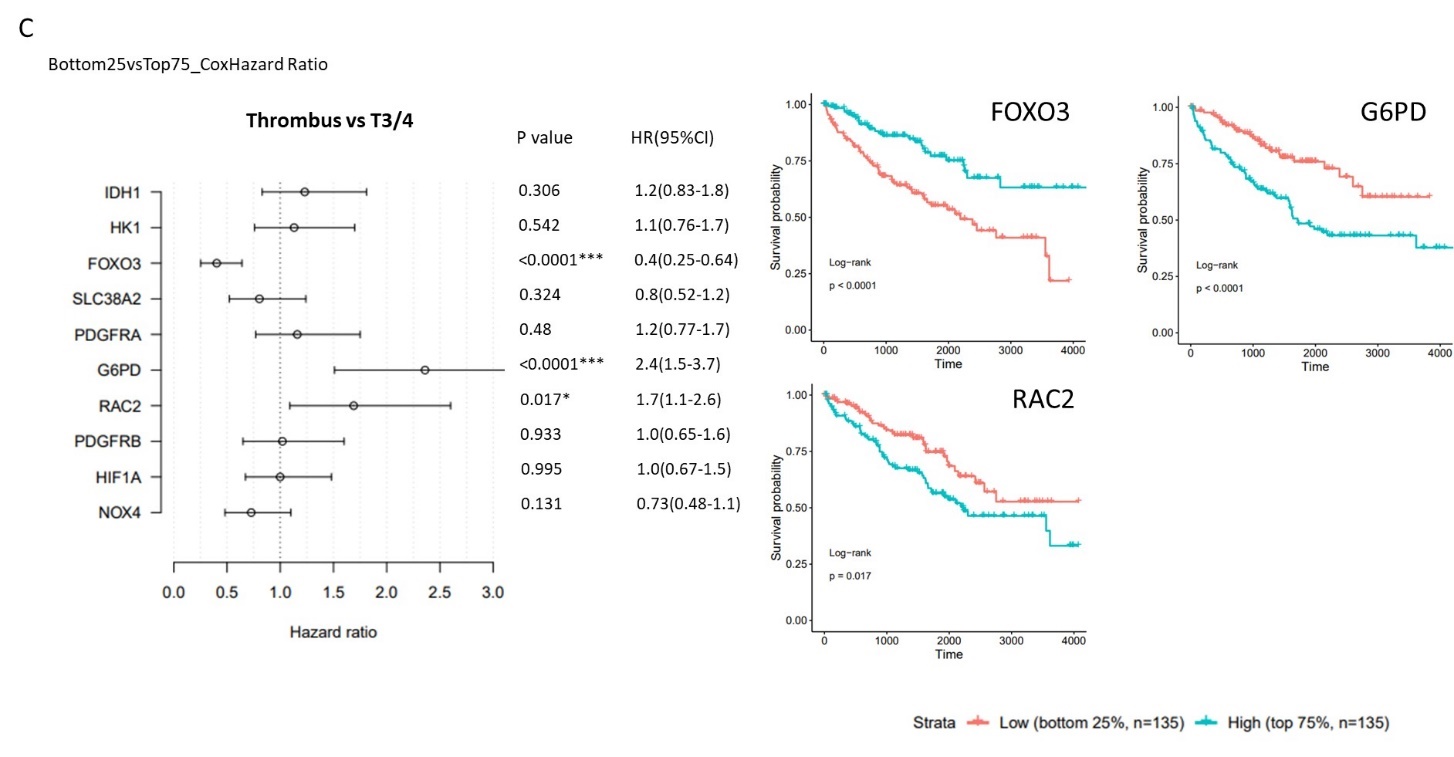


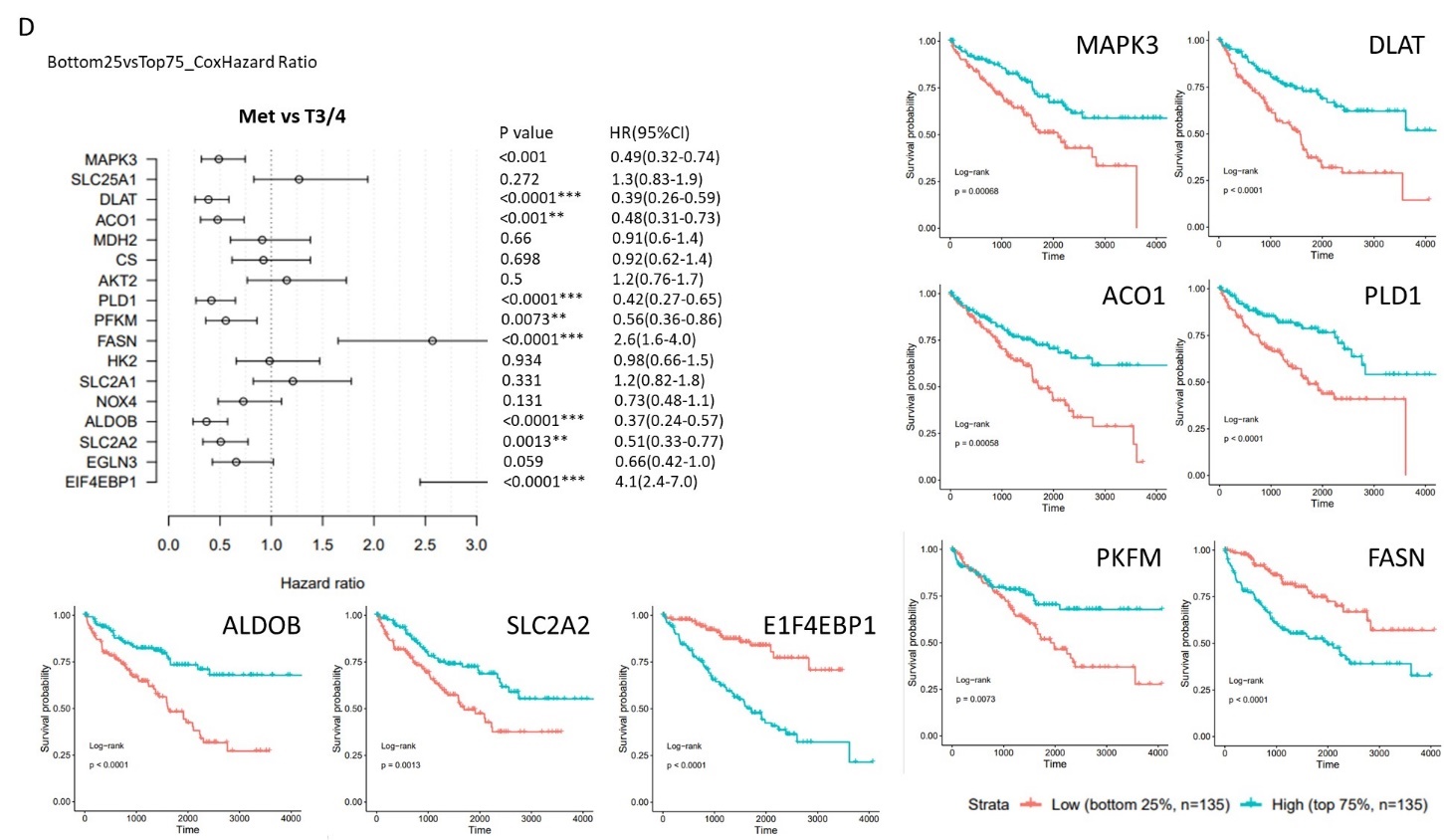


**Sup Figure 2: Metabolic genes associated with prognosis at different stages of renal cell carcinoma**

Association between gene expression of selected metabolic genes and overall survival of ccRCC patients in the TCGA-KIRC dataset. Univariate Cox regression and Kaplan-Meier overall survival of patients stratified according to gene expression as high (above 75%) and low (below 25%). Forest plot shows Hazard ratio (HR) and 95% confidence interval. Results for genes differentially expressed in A stage p1-2 relative to normal tissue, B state p3-4 relative to stage p1-2, C thrombi relative to stage p3-4, and D advanced metastatic stage relative to stage p3-4.
